# Profiling depression in childhood and adolescence: the role of conduct problems

**DOI:** 10.1111/jcpp.12465

**Published:** 2015-09-24

**Authors:** Lucy Riglin, Anita Thapar, Katherine H. Shelton, Kate Langley, Norah Frederickson, Frances Rice

**Affiliations:** ^1^ Institute of Psychological Medicine and Clinical NeurosciencesMRC Centre for Neuropsychiatric Genetics and GenomicsCardiff UniversityCardiffUK; ^2^ Department of ClinicalEducational and Health PsychologyUniversity College LondonLondonUK; ^3^ School of PsychologyCardiff UniversityCardiffUK

**Keywords:** Depression, aetiology, heterogeneity, conduct problems, genetic

## Abstract

**Background:**

Depression is typically more common in females and rates rise around puberty. However, studies of children and adolescents suggest that depression accompanied by conduct problems may represent a different subtype not characterised by a female preponderance, with differing risk factors and genetic architecture compared to pure‐depression. This study aimed to identify aetiologically distinct profiles of depressive symptoms, distinguished by the presence or absence of co‐occurring conduct problems.

**Methods:**

Latent profile analysis was conducted on a school sample of 1648 children (11–12 years) and replicated in a sample of 2006 twins (8–17 years).

**Results:**

In both samples pure‐depressive and conduct‐depressive profiles were identified. The pure‐depressive profile was associated with female gender, while the conduct‐depressive profile was associated with lower cognitive ability but not with gender. Twin analyses indicated possible differences in genetic aetiology.

**Conclusions:**

There was evidence for aetiologically heterogeneous depression symptom profiles based on the presence or absence of co‐occurring conduct problems.

## Introduction

Depression is a major contributor to the global burden of disease and is estimated to be the leading cause of disability worldwide (Lopez & Murray, 1998). Adolescence is an important period for the onset of depressive symptoms and disorder and both are associated with a range of debilitating outcomes including concurrent and future social and educational impairment, unemployment, poor physical health and mental health problems and suicidal behaviours in adult life (Thapar, Collishaw, Pine, & Thapar, 2012). Consistent with the view that depression is a complex multifactorial disorder (Rutter, 2007) there is considerable aetiological heterogeneity in child and adolescent depression (Rice, 2010). For instance, heritability estimates vary widely (Rice, Harold, & Thapar, 2002a) and both heterotypic and homotypic continuity with psychiatric disorders in adult life has been reported (Thapar et al., 2012). Furthermore, genome‐wide association studies have so far found no significant associations for major depressive disorder, which may be due in part to high heterogeneity in the disorder (Sullivan, Daly, & O'Donovan, 2012). This raises the possibility that there may be distinct subgroups of child and adolescent depression with differing aetiologies. One potential source of heterogeneity in childhood and adolescent depression is whether or not depressive symptoms co‐occur with conduct problems. Depressive symptoms and disorder are more common in girls compared to boys from midpuberty onwards (Thapar et al., 2012) but there is evidence that depression that co‐occurs with conduct problems does not show a female preponderance or the observed increase in rates during adolescence found for ‘pure’ depression (Angold & Rutter, 1992).

While it is known that depression is strongly associated with psychosocial adversity such as stressful life events and family conflict (e.g. Lewinsohn et al., 1994), clinical studies that directly compare depression with and without conduct problems have found some risk factors, including low IQ and adverse family environments (e.g. parental hostility and institutional/foster care), to be more strongly associated with depression that co‐occurs with conduct problems (Fombonne, Wostear, Cooper, Harrington, & Rutter, 2001a; Simic & Fombonne, 2001). Studies investigating trajectories of depressive symptoms and conduct problems have also found that this co‐occurrence is associated with greater family conflict and lower academic attainment than high levels of depressive symptoms alone (Chen & Simons‐Morton, 2009). Depression and conduct problems show longitudinal as well as cross‐sectional co‐occurrence: specifically, disruptive disorders in childhood and adolescence have been found to predict subsequent depression (Copeland, Shanahan, Costello, & Angold, 2009). Depressive symptoms have also been associated with particularly poor outcomes when they co‐occur with conduct problems, including low academic attainment, low social competence and substance use (Ingoldsby, Kohl, McMahon, & Lengua, 2006). Clinical observations suggest that when depression arises in children the presentation typically also involves conduct problems (Brent & Weersing, 2008). Unlike adolescent depression, childhood depression shows low levels of continuity with adult depression, instead showing heterotypic continuity with disorders such as conduct disorder and alcohol abuse/dependence (Weissman et al., 1999). Also unlike adolescent depression, childhood depression is equally common in boys and girls (Thapar et al., 2012). Finally, while there is strong evidence for an association between stress and depression (Hammen, 2005) there is some evidence that depressive disorder with an onset in childhood (where co‐occurring conduct problems are more likely) may be associated with different environmental stressors, specifically, greater adverse family factors, such as family discord and parental hostility compared to adolescent depression (Harrington et al., 1997). Taken together, this suggests aetiological heterogeneity and is consistent with the highly variable heritability estimates that have been reported from twin studies of childhood and adolescent depressive symptoms. One particularly reliable finding from twin studies is that genetic factors are less important in the aetiology of depressive symptoms in childhood but make a contribution to the aetiology of depressive symptoms in adolescence similar in magnitude to that seen for major depressive disorder in adults (Rice, Harold, & Thapar, 2002b). If depressive symptoms and disorder that co‐occur with conduct problems are more common in childhood and differ aetiologically from depressive symptoms without conduct problems, this may explain the age‐related genetic heterogeneity observed to date.

We used latent profile analysis to derive depressive subgroups and hypothesised: (a) depressive subgroups would be identified and defined by the absence and presence of co‐occurring conduct problems; (b) these profiles would show differing associations with risk factors tested as ‘validators’ of the profiles, specifically the pure‐depressive profile would be associated with female gender, while the conduct‐depressive profile would be associated with lower cognitive ability and parent hostility; (c) these profiles would also differ in genetic aetiology, with genetic factors less important in the aetiology of the conduct‐depressive compared to the pure‐depressive profile.

## Method

### Sample and design

#### School sample

1648 children aged 11–12 years old recruited from ten mixed, nonselective secondary schools, in South‐East England, UK. The sample, who participated in the second stage of the School Transition and Adjustment Research Study (STARS) in 2012 (Riglin et al., 2015) was broadly representative of the English population (DfE, 2011). Children were included in the present analyses when data on depressive symptoms were available. Parents were given the opportunity to opt their children out of the study and informed pupil assent was obtained.

#### Twin sample

2006 twins aged 8–17 years old: 424 monozygotic (MZ) twin pairs and 579 dizygotic (DZ) twin pairs. The twins, who had participated in The Greater Manchester Twin Register (Thapar, Harrington, Ross, & McGuffin, 2000), were born in Greater Manchester and Lancashire, England, between 1980 and 1991. Data on depressive symptoms were collected in 2000 and on conduct problems in 1996. Twins were included in analyses where depressive symptoms were available for both twins. Informed consent was obtained.

### Measures

#### Profile indicators

##### Depression

Depressive symptoms were measured using the self‐report form of the Short Mood and Feelings Questionnaire (SMFQ; Angold, Costello, Messer, & Pickles, 1995) in the school sample and the parent‐report form of the long version of the Moods and Feelings Questionnaire (MFQ; Costello & Angold, 1988) in the twin sample. The scales consist of 13 and 34 items respectively designed to cover core symptoms of DSM‐III‐R depression (APA, 1987). Items ask about symptoms during the past 3 months on a 3‐point scale: true (2); sometimes true (1) and not true (0), summed to produce a total score. Internal consistency was *α* = .89 and *α* = .93 for the SMFQ and MFQ respectively. Both self‐ and parent reports of depressive symptoms have been found to provide clinically useful information (Rice, Lyford, Thomas, & Thapar, 2007). Parent reports were used in the twin sample to allow comparability to previous twin studies (Rice et al., 2002b). Clinical cut‐points of 11 and 21 have been suggested for the SMFQ and MFQ respectively (Angold, Erkanli, Silberg, Eaves, & Costello, 2002; Wood, Kroll, Moore, & Harrington, 1995) which differentiate those with major depressive disorder from nondepressed children and adolescents. We used these to label which profiles included high levels of depressive symptoms, which also allowed comparability across the two samples.

##### Conduct problems

Conduct problems were measured using the 5‐item Strength and Difficulties Questionnaire subscale (Goodman, 1997) and 6 antisocial behaviour items from the Rutter A/B scales (Rutter, Tizard, & Whitmore, 1970) in the school and twin samples respectively. The measures are very similar, highly correlated and cover the key domains of conduct problems (Goodman, 1997). Symptoms were rated on a 3‐point scale: certainly true/certainly applies (2); sort of true/applies somewhat (1) and not true/doesn't apply (0); summed to produce a total score. The ratings of two informants were combined, as it had been suggested that multiple informants are required for a comprehensive assessment of child conduct problems, with each informant (child/parent/teacher) providing additional information (Loeber, Green, Lahey, & Stouthamer‐Loeber, 1989). Teacher reports were used in both samples, together with child or parent reports for the school and twin samples respectively, due to data availability. The highest teacher or child/parent score for each item was used to calculate the total score. Internal consistency was *α* = .65 and *α* = .82 for the school and twin sample respectively. For descriptive purposes, scores within the 90th percentile of the samples were considered high (5 in both samples).

#### Risk factors (school sample)

##### Cognitive ability

Intelligence quotient (IQ) was measured by the Cognitive Abilities Test (CAT3; Lohman et al., 2001), a standardised assessment which measures verbal reasoning, quantitative reasoning and nonverbal reasoning; internal consistency was *α* = .86. Teacher rated academic attainment were either National Curriculum levels (*N* = 1362) or International Middle Years levels (*N* = 197). Levels for English, Maths and Science were transformed into a continuous score, standardized by school and summed; internal consistency was *α* = .85.

##### Parent hostility

Parental hostility was measured using child ratings of maternal behaviour in the past month using the 4‐item Iowa Youth and Families Project (IYFP) Interaction Rating Scales subscale (Melby et al., 1993) on a 7‐point scale from never (1) to always (7), summed to produce a total score. Internal consistency was *α *= .79. Scores for children who had not been in touch with their mother in the last month were excluded.

### Data analysis

#### Identifying depressive subgroups

Latent profile analysis (LPA) was used to investigate possible depressive subgroups based on the presence or absence of conduct problems. LPA is a person‐centred approach that aims to group similar individuals into categories and is useful when data include heterogeneous groups of people. It aims to describe the associations between observed variables (in this case, depressive symptoms and conduct problems) using the smallest number of categories (Muthén & Muthén, 2000). Categories (profiles) with differing mean levels of the investigated variables may account for skewness in variables within the whole sample. Interpretation of whether profiles represent genuinely distinct categories or simply a single, non‐normal distribution, should be based on interpretation in the light of additional (validator) variables and theory (Muthén, 2006). LPA was conducted on depressive symptoms and conduct problems using a robust maximum likelihood parameter estimator in Mplus (Muthén & Muthén, 1998–2012). Further information about model selection is provided as an online appendix (Appendix S1).

#### Risk factors

Associations between hypothesised risk factors and the depressive profiles were examined in the school sample using R3STEP analysis, which predicts latent profile membership (all profiles relative to the normative profile) from the risk factors, using a multivariate approach. Profile probabilities (i.e. the probability of an individual being in each profile) are used to take into account profile measurement error. Investigating associations between profiles and risk factors after establishing the best profile solution corrects for biasing in standard errors that can arise if the risk factors are included in the same model as the LPA (Asparouhov & Muthén, 2013). Measures of IQ and academic attainment were highly correlated (*r *=* *.83) and therefore not simultaneously entered into the analyses. Analyses were conducted separately entering gender, parental hostility and either academic attainment or IQ. Associations between the depressive profiles and risk factors in the twin sample were analysed in Stata using a multinomial logistic regression with robust standard error estimators (Williams, 2000), as R3STEP analysis is not possible with multiple categorical latent variables, as is the case for the twin analysis (see Appendix S1).

#### Genetic aetiology

Twin data were used to estimate genetic (A), shared environment (C) and nonshared environment (E) influences of depressive symptoms in Mplus with chi‐square used to evaluate goodness of fit. For preliminary ACE models run on symptoms, depressive symptoms were log transformed to allow comparability to previous work (Rice et al., 2002b) and reduce skewness. For the depressive profiles, separate ACE models were run on the (continuous) profile probabilities for each of the four depressive profiles using a robust maximum likelihood parameter estimator. Using profile probabilities as the model parameters avoids classifying individuals into the most likely profile, which can result in biased estimates and standard errors (Muthén, Asparouhov, & Rebollo, 2006).

#### Types of depressive symptoms

Finally, we sought to investigate whether the conduct‐depressive profile and pure‐depressive profile exhibited different types of depressive symptoms. Exploratory analyses of differences in depressive symptoms endorsed in the pure‐depressive and conduct‐depressive profiles were conducted using logistic regression. The outcome variable was dummy coded conduct‐depressive profile, with the pure‐depressive profile as the reference group. The predictor variables were 12 depressive symptoms which mapped onto the DSM‐IV‐TR criteria for major depressive disorder (depressed mood; irritability; anhedonia; diminished appetite; insomnia; hypersomnia; psychomotor agitation; psychomotor retardation; fatigue; worthlessness; diminished concentration; thoughts of death or dying; APA, 2000), entered individually. Symptoms were coded as present if reported to be at least sometimes true. A robust standard error estimator was used for the twin sample.

## Results

Descriptive statistics and correlations between variables are presented in Table 1. Depressive symptoms and conduct problems were correlated with each other and with all risk factors, with the exception that age was not associated with depressive symptoms in the twin sample. Depressive symptoms were associated with female gender and conduct problems were associated with male gender in both samples.

**Table 1:** Descriptive statistics and correlations between variables

(A) School sample (*N* = 1648)	1.	2.	3.	4.	5.	6.	
1. Depressive symptoms (SMFQ)							
2. Conduct problems symptoms	**.370**						
3. Gender^a^	**.051**	**−.242**					
4. IQ	**−.088**	**−.218**	.010				
5. Academic attainment	**−.112**	**−.268**	**.079**	**.831**			
6. Maternal hostility	**.434**	**.371**	**−.066**	**−**.017	**−.101**		
Mean (*SD*)	4.024 (4.670)	2.055 (1.915)	.470 (.499)	102.172 (11.834)	.004 (.872)	10.623 (4.983)	

(B) Twin sample (*N* = 2008)	1.	2.	3.				7.
1. Depressive symptoms (MFQ)							
2. Conduct problems symptoms	**.241**						
3. Gender^a^	**.054**	**−.164**					
7. Age (months)	.038	**−.116**	.021				
Mean (*SD*)	9.155 (8.926)	1.821 (2.331)	.537 (.499)				155.082 (28.484)

Significant correlations at *p *<* *.05 indicated in bold. SMFQ, Short Mood and Feelings Questionnaire; MFQ, Moods and Feelings Questionnaire (long version).

a 0 = boys, 1 = girls.

### Latent profiles

**Figure 1:**
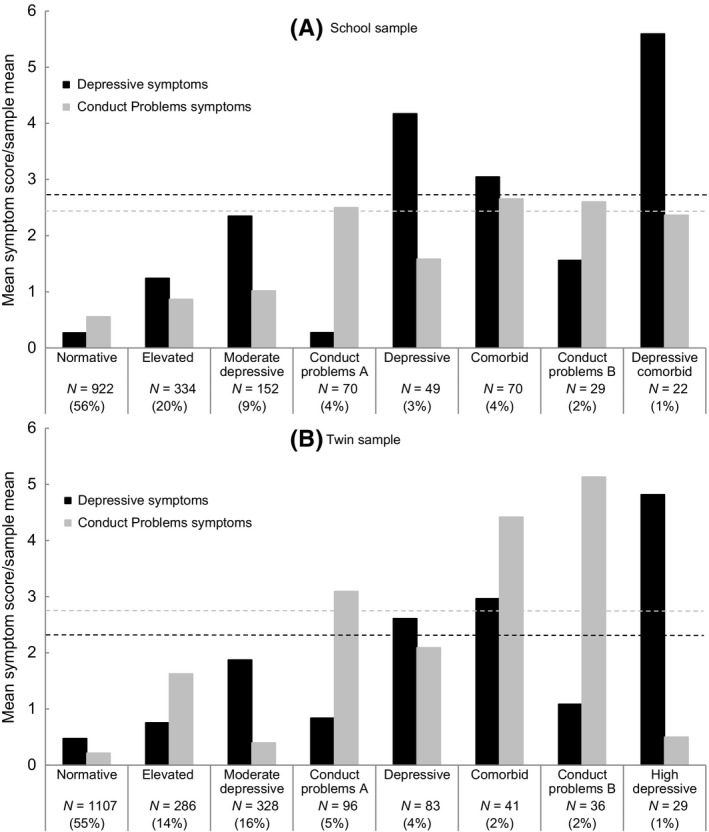
Depressive symptoms and conduct problems for the eight profiles. Mean depressive symptoms and conduct problems for each profile are presented divided by the sample mean for comparability across samples. Dashed lines represent the equivalent suggested clinical cut‐points for depressive symptoms (11 for the school sample and 21(Angold et al., 2002; Wood et al., 1995) for the twin sample) and the 90th percentile for conduct problems

In both samples latent profile analysis identified eight profiles (see Appendix S1). These were two profiles with relatively low levels of all symptoms, two characterised by conduct symptoms without depressive symptoms and four characterised by depressive symptoms (with and without co‐occurring conduct problems). Figure 1 shows mean depressive symptoms and conduct problems for each profile (for comparability, profile means are presented divided by the sample mean). Results described in text focus on the depressive profiles given our hypotheses. In both samples the depressive profiles were: moderate depressive; depressive; comorbid; depressive comorbid/high depressive. The moderate‐depressive profile (9% and 16% prevalence in the school and twin sample respectively) was defined by moderate levels of depressive symptoms in the absence of conduct symptoms. The depressive profile (3% and 4%) was defined by mean level depressive symptoms which met a clinical cut‐point in the absence of conduct symptoms. The comorbid profile (4% and 2%) was defined by mean level depressive symptoms which met a clinical cut‐point with co‐occurring high symptoms of conduct problems. Finally, the fourth profile (1% prevalence in both samples) was defined by particularly high levels of depressive symptoms, scoring more than double the clinical cut‐points. In the school sample this was accompanied by co‐occurring moderate symptoms of conduct problems and therefore labelled depressive comorbid, whereas in the twin sample it was not accompanied by symptoms of conduct problems and thus labelled high depressive.

Associations between these profiles and risk factors are given in Table 2. The moderate‐depressive, depressive and high‐depressive profiles included more girls than boys (school sample: 57%–61% female; twin sample: 53%–68%), whereas the comorbid and depressive‐comorbid profiles did not (school sample: 43%–47% female; twin sample: 49%). Multivariate analyses presented in Table 2 indicated that: (a) female gender predicted membership of the moderate‐depressive and depressive profiles in the school sample and somewhat predicted the moderate‐depressive profile in the twin sample; (b) low attainment and IQ predicted membership of the comorbid profile; (c) low attainment also predicted membership of the moderate‐depressive profile; (d) maternal hostility predicted membership of all depressive profiles; (e) in the twin sample, older age predicted membership of the high‐depressive profile and there was a trend for a relationship between younger age and membership of the comorbid profile.

**Table 2:** Associations between depressive profiles and risk factors

A: School sample (*N* = 1504)	Moderatedepressive (9%) B (*SE*)	Depressive (3%) B (*SE*)	Comorbid (4%) B (*SE*)	Depressive comorbid (1%) B (*SE*)
Gender proportions	57% female	61% female	47% female	43% female
Gender^a^	.480 (.220)*	.708 (.338)*	**−**.024 (.349)	**−**.225 (.541)
Attainment	**−**.405 (.130)**	**−**.090 (.222)	**−**.903 (.209)***	**−**.228 (.363)
IQ^b^	**−**.023 (.013)	.012 (.024)	**−**.081 (.022)***	**−**.041 (.026)
Maternal hostility	.215 (.024)***	.309 (.035)***	.216 (.034)***	.483 (.061)***

B: Twin sample (*N* = 2001)	Moderate depressive (16%) B (*SE*)	Depressive (4%) B (*SE*)	Comorbid (2%) B (*SE*)	High depressive (1%) B (*SE*)
Gender proportions	62% female	53% female	49% female	68% female
Gender^a^	.260 (.148)^†^	**−**.102 (.247)	**−**.268 (.327)	.517 (.459)
Age (months)	.002 (.003)	**−**.004 (.004)	**−**.013 (.007)^†^	.016 (.008)*

The normative profile is the reference group, analyses included all 8 profiles.

a 0 = boys 1 = girls.

b IQ was included in a separate model (*N* = 1016) to attainment as these were highly correlated (*r *=* *.831) results for gender and maternal hostility revealed the same pattern of results in both models, with the exception that gender was no longer significantly associated with the moderate‐depressive profile for the IQ model, although the estimates were similar (B = .449, *SE *= .279; result available from the first author).

^†^ *p *<* *.1; *p <* *.05; ** *p *<* *.01; *** *p *<* *.001.

### Genetic contributions to depressive symptoms

Genetic model fitting results and concordance rates are given in Table 3. For depressive symptoms, results indicated the contribution of genetic, shared environment and nonshared environment factors were 25%, 51% and 24% respectively, in line with previous reports that included the current sample plus additional participants (Rice et al., 2002b). For the depressive profiles, estimated genetic influence was modest for the moderate‐depressive and conduct‐depressive profiles (32% and 17% respectively), but larger for the pure‐depressive profile and the high‐depressive profiles (52% and 54% respectively). Larger shared environmental influence was estimated for the moderate‐depressive profile (46%) compared to the pure‐depressive, conduct‐depressive and high‐depressive profiles (0%). Finally, nonshared environment estimated influence was largest for the conduct‐depressive profile (83%) compared to the moderate‐depressive, pure‐depressive and high‐depressive profiles (22%, 48% and 46% respectively). Thus, there was some indication that genetic factors were most important for the pure‐depressive and high‐depressive profiles, shared environment for the moderate‐depressive profile and nonshared environment for the conduct‐depressive profile. Finally, we restricted analyses to older twins (aged 11–17; *N* = 1542) given reports that the genetic contribution to depressive symptomatology is greater in this age group. Genetic influence was estimated to be 23% in the moderate‐depressive profile, 48% in the pure‐depressive profile, 6% in the conduct‐depressive profile and 47% in the high‐depressive profile.

**Table 3:** Genetic model fitting

	Twin pair correlations		Parameter estimates (% with 95% CI)			Model fit	
	MZ	DZ	A	C	E	*χ*2 (*df* = 6)	
Depressive symptoms							
Whole sample *N* = 2006	.763	.627	25 (11–38)	51 (39–63)	24 (20–29)	3.629	*p *= .727
Profiles *N* = 2006							
Moderate‐depressive	.775	.641	32 (1–63)	46 (22–72)	22 (11–32)	8.726	*p *= .190
Pure‐depressive	.527	.201	52 (29–74)	0 (0–0)	48 (26–71)	0.872	*p *= .990
Conduct‐depressive	.222	−.002	17 (0–43)	0 (0–0)	83 (57–100)	4.737	*p *= .578
High‐depressive	.568	.115	54 (5–100)	0 (0–0)	46 (0–95)	6.870	*p *= .330

	Number affected		Proband concordance rate	
	MZ	DZ	MZ	DZ
Categorical profiles				
Moderate‐depressive	132	196	.803	.694
Pure‐depressive	37	46	.432	.174
Conduct‐depressive	15	26	.133	0
High‐depressive	11	18	.545	.111

Twin pair correlations and parameter estimates are based on (continuous) profile probabilities for the whole sample; number affected and proband concordance rates are based on categorical profiles. Parameter estimates for all profiles are based on full ACE models.

### Depressive symptoms

The only significant predictor of the conduct‐depressive compared to the pure‐depressive profile was fewer self‐reported thoughts of death or dying in the school sample [Odds Ratio (OR) = .335, *p *<* *.05], which was also found at trend level for self‐reported symptoms in the twin sample (OR = .388, *p *<* *.1). No parent‐reported symptoms were significant.

## Discussion

This study aimed to identify aetiologically distinct depressive profiles based on co‐occurrence with conduct symptoms. It was predicted that pure‐depressive and conduct‐depressive profiles would be identified, defined by the absence and presence of co‐occurring conduct problems respectively. It was hypothesised that these profiles would differ in risk factors and genetic aetiology.

### Pure‐depressive and conduct‐depressive profiles

The results from both samples support the first hypothesis in that distinct pure‐depressive (prevalence estimates 3%–4%) and conduct‐depressive (prevalence estimates 2%–4%) profiles were identified. The results also generally supported the second hypothesis: (a) female gender was associated with the pure‐depressive profile but not the conduct‐depressive profile, (b) lower IQ and academic attainment were associated with the conduct‐depressive but not the pure‐depressive profile (parent hostility was associated with both profiles). These results comparing risk factors for pure and conduct‐depressive profiles therefore show similarity with those reported in clinical samples (Angold & Rutter, 1992; Fombonne et al., 2001a). If depression accompanied by conduct problems is the more common presentation in boys, lower IQ may partially explain reports of worse functional outcomes for depressed boys than depressed girls (Dunn & Goodyer, 2006). Future work assessing the long‐term outcomes associated with the different depressive profiles, and the potential role of low IQ, is needed to investigate this suggestion.

In support of the third hypothesis, there was preliminary evidence of differences in genetic aetiology. Genetic factors were estimated to be less important for the conduct‐depressive than the pure‐depressive profile (17% and 52% respectively), which instead was estimated to have greater nonshared environmental influence (83%; although confidence intervals were overlapping). One possible explanation is that the conduct‐depressive profile included younger twins. Indeed, conduct problems typically show a younger age of onset than depressive symptoms (Kessler et al., 2005) and the conduct‐depressive profile was somewhat associated with younger age. However, genetic models for older twins only gave the same pattern of results. A larger proportion of depressive symptoms with co‐occurring conduct problems in childhood compared to adolescence, may partially explain why genetic factors are found to be less important during childhood (Rice et al., 2002b). Further research is needed to investigate why conduct‐depression might be less heritable than pure‐depression. For the conduct‐depressive profile, nonshared environmental risk factors appear to play a particularly important role. These are environmental factors that serve to make twins dissimilar from each other and might include differential treatment by parents and peers.

Finally, our exploratory analyses found some evidence of differences in the type of depressive symptoms exhibited in the conduct‐depressive compared to pure‐depressive profile: specifically, fewer (self‐rated) thoughts of death and dying. This differs from a clinical follow‐up study of children with major depressive disorder which found increased suicide attempts for those with conduct disorder than without (Fombonne, Wostear, Cooper, Harrington, & Rutter, 2001b). However, thoughts of death and dying may not be equivalent to suicide attempts. Our findings are consistent with work on adult populations which suggests that core cognitive symptoms including suicide ideation may have separate genetic and environmental influences to core mood symptoms such as low mood and anhedonia (Kendler, Aggen, & Neale, 2013). Again, our work requires replication to establish whether different types of depressive symptoms are present in conduct‐depression to pure‐depression and whether these differences may result in the observed differences in genetic aetiology.

In summary, our findings suggest that heterogeneity in the aetiology of childhood and adolescent depressive symptoms and disorder may be partially due to the presence or absence of co‐occurring conduct problems. This has implications for nosology given that ICD‐10 and DSM‐V classify depression with conduct disorder differently. While we find evidence of aetiological differences, further research is required to investigate the validity of considering pure and conduct‐depressive disorders separately (e.g. Fombonne et al., 2001a; Taylor & Rutter, 2008).

### Additional depressive profiles: symptom severity

Two additional depressive profiles were identified in both samples which were differentiated by depressive symptom severity rather than the presence or absence of conduct problems. Firstly, a ‘subthreshold’ depressive profile was somewhat similar to the pure‐depressive profile in being associated with female gender and not with IQ. However, this profile was associated with low academic attainment, which supports the importance of addressing subthreshold symptoms (Angold, Costello, Farmer, Burns, & Erkanli, 1999; Thapar et al., 2012). This also suggests that depressive symptoms are associated with a risk of low academic attainment in the absence of conduct problems, consistent with a recent meta‐analysis which found depressive symptoms to be associated with subsequent school grades (Riglin, Petrides, Frederickson, & Rice, 2014). Prevalence rates for this profile differed between the school and twin samples (9% and 16% respectively), which is likely due to the differing age ranges, with the latter including more adolescents (Thapar et al., 2012). Secondly, a profile with very high‐depressive symptoms was identified, comprising 1% of participants. This was an additional conduct‐depressive profile in the school sample but a pure‐depressive profile in the twin sample. This difference may be due to the age differences of the samples.

We also found evidence of genetic heterogeneity based on symptom severity. Specifically, lower levels of depressive symptoms were estimated to have a smaller genetic influence than those with higher depressive symptoms (32% in the moderate‐depressive compared to 52%–54% in the pure/high‐depressive profiles; although confidence intervals overlapped) and larger shared environmental influences (46% compared to 0%). A similar finding was reported in a previous study of young people, which found shared environmental influences for a broad phenotype of sadness and/or anhedonia but not for major depressive disorder (Glowinski, Madden, Bucholz, Lynskey, & Heath, 2003). Twin studies of hospital ascertained major depressive disorder in adults also report high heritability estimates consistent with the idea that severity may be an important influence on the magnitude of genetic estimates (McGuffin, Katz, Watkins, & Rutherford, 1996; Thapar et al., 2012). However, high‐depressive symptom scores in children and adolescents have tended to be influenced by shared environmental factors rather than genetic factors (Rice, 2010). Our findings should be considered exploratory because of the wide and overlapping confidence intervals and replication is required, but they indicate that there is likely to be heterogeneity in high symptom scores based on the co‐occurrence of conduct problems. Thus, our findings suggest two possible sources of variation in heritability estimates: a) the co‐occurrence of conduct problems, which leads to reduced heritability estimates and increased estimates of nonshared environment, and, b) symptom severity, with lower levels of symptoms leading to reduced heritability estimates and increased estimates of shared environment.

Limitations and issues worth considering are differences between the two samples, the use of questionnaires to assess symptoms and small informative groups for some rarer profiles. While the latent profiles were broadly consistent across the two samples, a number of sample differences may have influenced results. First, data on symptoms of conduct problems were collected roughly 4 years before the depressive symptoms in the twin sample but concurrently in the school sample. Secondly, depressive symptoms were self‐reported in the school sample and parent‐reported in the twin sample (parent ratings were used for twin analyses because these show age‐related variation in genetic aetiology). Finally, the two samples included different age ranges. Nevertheless, the observed distinct pure‐ and conduct‐depressive profiles were identified in both samples. Questionnaires enabled data to be collected from two large, representative samples and subthreshold symptoms to be investigated but the depressive profiles identified may not extend to clinical disorders. Analyses indicated an 8‐profile solution, which resulted in some rare profiles that included a small number of informative individuals (see below). Nevertheless, a number of recommended criteria indicated that at least eight profiles were required to describe associations between depressive symptoms and conduct problems in both samples. Differing associations with risk factors also validates these as distinct profiles (Muthén, 2006). Analyses were based on largely unaffected community samples meaning that despite the relatively large sample sizes (1648 and 2006), analysis of risk factors and genetic aetiology were limited in power due to the relatively small sizes of the depressive profiles (1%–4% for the pure/comorbid‐depressive profiles). This is likely to have been an issue for the high‐depressive profile. It is also important to note that the confidence intervals for genetic and environmental estimates for pure and conduct‐depressive profiles overlapped. Thus, while our observations provide some insight into heterogeneity in risk factors and the genetic aetiology of depressive symptoms, they merit replication. Finally, it is possible that the profiles identified are specific to the developmental period investigated. Future work could investigate the stability of these profiles, whether they replicate at different ages and the sequence of presentation of depressive and conduct symptoms in the conduct‐depressive profiles, which would also likely show heterogeneity.

## Conclusion

This study indicates that there are distinct depressive profiles distinguished by the presence or absence of co‐occurring conduct problems. In a school sample, a pure‐depressive profile was associated with female preponderance, whereas conduct‐depressive profiles were associated with low IQ and poor academic attainment. Both profiles were associated with higher parent hostility and a ‘subthreshold’ depressive profile was associated with female gender and poor academic attainment. The pure‐depressive and conduct‐depressive profiles were replicated in a twin sample, which found some evidence of different genetic aetiologies. Findings suggest that inconsistencies in risk factors, heritability estimates and outcomes of childhood and adolescent depressive symptoms and disorder may be due to the presence of different depressive subgroups.

**Key points**

- Studies of children and adolescents suggest that depression accompanied by conduct problems may represent a different subtype not characterised by a female preponderance, with differing risk factors and genetic architecture compared to pure‐depression.
- In two independent samples, latent profile analysis identified pure‐depressive and conduct‐depressive profiles.
- The pure‐depressive profile was associated with female gender, while the conduct‐depressive profile was associated with lower cognitive ability but not gender. Twin analyses indicated possible differences in genetic aetiology.
- Inconsistencies in risk factors, heritability estimates and outcomes of child and adolescent depressive symptoms and disorder may be due to the presence of different depressive subgroups.

## Supporting information


**Appendix S1.** Latent profile analysis model selection.
**Table S1.** Model comparisons for latent profile analyses.
