# Supplementary material for: Profiling depression in childhood and adolescence: the role of conduct problems
Source: J Child Psychol Psychiatry. 2015 Sep 24;57(4):481–90. doi: 10.1111/jcpp.12465 (PMC5102656; doi:10.1111/jcpp.12465)
Supplement: Supplementary file 1 — Appendix S1. Latent profile analysis model selection. Table S1. Model comparisons for latent profile analyses. [file JCPP-57-481-s001.docx]

**Supporting information for *Profiling depression in childhood and adolescence: the role of conduct problems* by Riglin et al.**

**Appendix S1: Latent profile analysis model selection**

Latent profile analysis aims to group similar individuals into categories. Starting with a single *k*-profile solution, *k*+1 solutions were extracted until the optimum number of profiles was reached (i.e. the fewest number of profiles that describe associations between depressive symptoms and conduct problems). As recommended, a number of criteria were used to determine the optimum number of profiles: 1) improved model fit for *k*+1 compared to *k* solutions according to the bootstrap likelihood ratio test (BLRT) or a robust chi-square difference test (for twin data; see below) and the Bayesian information criterion (BIC)([Nylund, Asparouhov, & Muthen, 2008](#_ENREF_4)); 2) high entropy values ([>=.80; Clark & Muthén, 2009](#_ENREF_1)); 3) the extent to which profiles were theoretically meaningful ([Muthen & Muthen, 2000](#_ENREF_3)). For fitting latent profile models to twin data, a two-level approach was used which accounts for twin non-independence. This involves modelling two latent profile variables (one for ‘Twin a’ and one for ‘Twin b’, to which individuals from each twin pair were randomly assigned) for which the parameters are held equal but where the probabilities for Twin a are not influenced by the probabilities for Twin b and vice versa ([Muthen, Asparouhov, & Rebollo, 2006](#_ENREF_2)). The BLRT cannot be computed for two-levels models, instead a robust chi-square difference test was used ([Satorra & Bentler, 2001](#_ENREF_5)).

Model comparisons for both samples are shown in Table S1. In both samples, a significant improvement in model fit from 1 to 9 profiles was indicated by BLTR or χ^2^ change. However BIC and loglikelihood values evened out from 8 to 9 profiles. This suggests that fewer than 8 profiles did not adequately describe associations between depressive symptoms and conduct problems in either sample. Thus an 8 profile solution was selected for both samples.

**Table S1: Model comparisons for latent profile analyses**

| Profiles | Free parameters | Loglikelihood value | BIC | Entropy | BLTR: 2 times the loglikelihood difference |
| --- | --- | --- | --- | --- | --- |
| *School sample* | | | | | |
| 1 | 6 | -6751.379 | 13532.387 |  |  |
| 2 | 10 | -6417.843 | 12887.538 | .912 | 667.071^*^ |
| 3 | 14 | -6272.889 | 12619.850 | .912 | 289.909^*^ |
| 4 | 18 | -6187.190 | 12470.675 | .888 | 171.398^*^ |
| 5 | 22 | -6126.065 | 12370.647 | .913 | 122.249^*^ |
| 6 | 26 | -6083.867 | 12308.473 | .908 | 84.396^*^ |
| 7 | 30 | -6054.291 | 12271.542 | .856 | 59.152^*^ |
| 8 | 34 | -6022.602 | 12230.387 | .835 | 63.377^*^ |
| 9 | 38 | -6005.211 | 12217.828 | .840 | 34.782^*^ |
| Profiles | Free parameters | Loglikelihood value | BIC | Entropy | Satorra-Bentler Scaled Chi-Square |
|  | | | | | |
| 1 | 8 | -10557.399 | 21170.083 |  |  |
| 2 | 10 | -10215.965 | 20501.037 | .878 | 146.746^*^ |
| 3 | 15 | -10007.167 | 20117.995 | .858 | 193.099^*^ |
| 4 | 21 | -9829.090 | 19803.305 | .816 | 425.869^*^ |
| 5 | 28 | -9721.909 | 19637.318 | .818 | 33.965^*^ |
| 6 | 36 | -9610.104 | 19468.995 | .833 | -139.997^#^ |
| 7 | 45 | -9528.773 | 19368.530 | .823 | -1047.405^#^ |
| 8 | 55 | -9455.856 | 19291.803 | .830 | 223.929^*^ |
| 9 | 66 | -9412.587 | 19281.284 | .828 | 69.087^*^ |

^*^*p*<.0001 ^#^ *p* values not computed for negative values. BIC = Bayesian Information Criterion, BLTR = bootstrap likelihood ratio test.

**References**

Clark, S. L., & Muthén, B. (2009). Relating latent class analysis results to variables not included in the analysis. *Submitted for publication*.

Muthen, B., Asparouhov, T., & Rebollo, I. (2006). Advances in behavioral genetics modeling using Mplus: Applications of factor mixture modeling to twin data. *Twin Research and Human Genetics, 9*, 313-324.

Muthen, B., & Muthen, L. K. (2000). Integrating person-centered and variable-centered analyses: Growth mixture modeling with latent trajectory classes. *Alcoholism-Clinical and Experimental Research, 24*, 882-891.

Nylund, K. L., Asparouhov, T., & Muthen, B. O. (2008). Deciding on the number of classes in latent class analysis and growth mixture modeling: A Monte Carlo simulation study.

Satorra, A., & Bentler, P. (2001). A scaled difference chi-square test statistic for moment structure analysis. *Psychometrika, 66*, 507-514.
